# VDACs Post-Translational Modifications Discovery by Mass Spectrometry: Impact on Their Hub Function

**DOI:** 10.3390/ijms222312833

**Published:** 2021-11-27

**Authors:** Maria Gaetana Giovanna Pittalà, Stefano Conti Nibali, Simona Reina, Vincenzo Cunsolo, Antonella Di Francesco, Vito De Pinto, Angela Messina, Salvatore Foti, Rosaria Saletti

**Affiliations:** 1 Molecular Biology Laboratory, Department of Biological, Geological and Environmental Sciences, University of Catania, Via S. Sofia 64, 95123 Catania, Italy; marinella.pitt@virgilio.it (M.G.G.P.); mess@unict.it (A.M.); 2 Department of Biomedical and Biotechnological Sciences, University of Catania, Via S. Sofia 64, 95123 Catania, Italy; stefano.continibali@unict.it (S.C.N.); simona.reina@unict.it (S.R.); vdpbiofa@unict.it (V.D.P.); 3 Organic Mass Spectrometry Laboratory, Department of Chemical Sciences, University of Catania, Via S. Sofia 64, 95123 Catania, Italy; vcunsolo@unict.it (V.C.); antonelladfrancesco@gmail.com (A.D.F.); sfoti@unict.it (S.F.)

**Keywords:** voltage dependent anion channel, cysteine overoxidation, deamidation, hydroxyapatite, high-resolution mass spectrometry, post-translational modifications

## Abstract

VDAC (voltage-dependent anion selective channel) proteins, also known as mitochondrial porins, are the most abundant proteins of the outer mitochondrial membrane (OMM), where they play a vital role in various cellular processes, in the regulation of metabolism, and in survival pathways. There is increasing consensus about their function as a cellular hub, connecting bioenergetics functions to the rest of the cell. The structural characterization of VDACs presents challenging issues due to their very high hydrophobicity, low solubility, the difficulty to separate them from other mitochondrial proteins of similar hydrophobicity and the practical impossibility to isolate each single isoform. Consequently, it is necessary to analyze them as components of a relatively complex mixture. Due to the experimental difficulties in their structural characterization, post-translational modifications (PTMs) of VDAC proteins represent a little explored field. Only in recent years, the increasing number of tools aimed at identifying and quantifying PTMs has allowed to increase our knowledge in this field and in the mechanisms that regulate functions and interactions of mitochondrial porins. In particular, the development of nano-reversed phase ultra-high performance liquid chromatography (nanoRP-UHPLC) and ultra-sensitive high-resolution mass spectrometry (HRMS) methods has played a key role in this field. The findings obtained on VDAC PTMs using such methodologies, which permitted an in-depth characterization of these very hydrophobic trans-membrane pore proteins, are summarized in this review.

## 1. Introduction

### 1.1. VDAC Isoforms: A Family of Hub Proteins

VDAC (voltage-dependent anion selective channel) proteins, also known as mitochondrial porins, are the most abundant proteins of the outer mitochondrial membrane (OMM) where they play a vital role in various cellular processes, in the regulation of metabolism, and in survival pathways. They mediate the ions and metabolites exchange between mitochondria and the rest of the cell, ensuring good functionality of mitochondrial complexes and energy production [1].

In higher eukaryotes, there are three VDAC isoforms (VDAC1, VDAC2, VDAC3) encoded by separate genes located on different chromosomes [2]. These pore-forming proteins have a similar molecular weight (30 kDa) and highly conserved sequences of about 280 amino acids with the exception of VDAC2 which has the N-terminal moiety of 11 residues longer than the other isoforms.

The evolutionary analysis indicates VDAC3 as the oldest isoform, while VDAC1 is considered the youngest mitochondrial porin [3,4].

The experimental 3D structures of mouse and human VDAC1 isoform have been determined using X-ray crystallography and NMR [5,6,7]. These analyses revealed a structure constituted by 19 β-strands arranged to form a trans-membrane β-barrel and by a region containing α-helix at the N-terminus of the protein. The barrel is organized as a regular antiparallel array of β-strands with the exception of strands 1 and 19 that run in parallel. The amphipathic α-helix tail is located inside the pore. However, the exact position and local structure of this segment are still elusive since these features are not perfectly overlapping in the available X-ray and NMR structures [5,6,7].

Recently, the structure of zebrafish VDAC2 was solved at a high resolution, confirming the same β-barrel arrangement as VDAC1 [8]. Zebrafish VDAC2 has one cysteine residue in the sequence and lacks the 11 amino acid longer N-terminal sequence present in mammalian VDACs.

The VDAC3 structure has not yet been determined. Several bioinformatic predictions, based on the large sequence similarity, proposed a barrel core such as the other VDAC isoforms [9]. Despite the high sequence similarity and structural homology, VDAC isoforms display different functional properties within the cell.

Analysis of the expression levels of human VDAC isoforms in HeLa cells, determined by real-time PCR, suggests that VDAC1 is the most abundant isoform, ten times more abundant compared to VDAC2 and hundred times more abundant compared to VDAC3, the least characterized of the isoforms. In addition, the overexpression of each single VDAC isoform affects the mRNA levels of the other two isoforms, suggesting that the ratios between VDAC isoforms are subjected to a reciprocal control that avoids an imbalance among these proteins [10].

Although the three isoforms show a common involvement in cellular bioenergetics maintenance, VDAC1 and VDAC2 have specialized functions in programmed cell death. For VDAC3 isoform, recent studies indicate a central role in ROS metabolism and in mitochondrial quality control [11].

The functions of VDACs are several-fold and some of these depend on, or are affected by, interaction with other cytosolic and mitochondrial proteins. Due to their localization at the OMM, VDACs are considered to be hub proteins, interacting with over 200 proteins in order to integrate mitochondrial functions with the rest of the cellular activities [12,13]. Thus, VDAC isoforms appears to be a junction for a variety of signals associated with different pathways related to cell survival or programmed death. Furthermore, the function of VDACs and their interactions with other proteins are affected by post-translational modifications (PTMs) [14]. Unfortunately, PTMs of VDAC proteins represent a little explored field, mainly because discovery and characterization of PTM in these proteins is very challenging, due to their poor solubility and impossibility to isolate single isoforms. Only in recent years has the increasing number of tools aimed at identifying and quantifying PTMs increased, improving the knowledge in this field and in the mechanisms that regulate functions and interactions of mitochondrial porins. In particular, the development of nano-reversed phase ultra-high-performance liquid chromatography (nanoRP-UHPLC) and ultra-sensitive high-resolution mass spectrometry (HRMS) methods has played a key role in this field. The findings obtained on VDAC PTMs using such methodologies, which have permitted an in-depth characterization of these very hydrophobic trans-membrane pore proteins, are summarized in this review.

### 1.2. VDACs as Main Players in Mediating and Regulating Mitochondrial Functions with Cellular Activities

The location in the OMM allows the VDAC proteins to act as anchor points for diverse sets of molecules that interact with mitochondria. In this way, VDACs are able to mediate and regulate the integration of mitochondrial functions with cellular activities.

The VDAC interactome includes proteins located in OMM, inner mitochondrial membrane (IMM), intermembrane space (IMS), cytosol, endoplasmic reticulum, plasma membrane and nucleus that are involved in metabolism, apoptosis, signal transduction, protection against ROS, binding to RNA or DNA, and more.

Mobility of VDAC N-terminal α-helix region is important for channel gating but also for interactions with both pro-apoptotic and anti-apoptotic proteins such as Bax, Bak, and Bcl-xL [15,16,17]. VDAC1 is involved in the release of apoptotic factors located in the intermembrane space due to its ability of oligomerizing in dimers, hexamers, and higher-order structures, to form a large pore that allows the passage of cytochrome c and apoptosis inducing factor (AIF) to the cytosol and consequently the activation of programmed cell death. Instead, VDAC2 functions as anti-apoptotic factor and it is upregulated in several debilitating diseases including Alzheimer’s and cancer [18]. This property is probably due to the unique ability of VDAC2 to sequester the pro-apoptotic protein Bak in the OMM and maintain it in the inactive state [11].

VDAC1 displays binding sites, located in its cytosolic moiety, for many metabolic enzymes, such as glyceraldehyde 3-phosphate dehydrogenase, creatine kinase, glycerol kinase, glucokinase, c-Raf kinase, and hexokinase isoforms (I and II), which need preferential access to mitochondrial ATP [19].

Hexokinase interacts through its hydrophobic N-terminal sequence with Glu^73^ of VDAC1, a binding site localized on one side of the barrel wall, buried in the hydrophobic environment of OMM [20].

It has been demonstrated that treatment of mitochondria with dicyclohexylcarbodiimide (DCCD) inhibits hexokinase–VDAC interaction due to selective chemical modification of Glu^73^ [21].

Glu^73^ residue is also the binding site for ceramides, tumor suppressor lipids able to act directly on mitochondria to trigger apoptotic cell death. It is interesting that both VDAC1 and 2 own, in a similar position, a cysteine residue (Cys^127^ in human VDAC1 and Cys^138^ in human VDAC2) in the form of sulfonic acid with a strong negative charge resembling that of the glutamate acid residue [22]. Instead, VDAC3 isoform does not show any residue homologous to Cys^127/138^ or Glu^73^ embedded in the hydrophobic moiety of the OMM.

VDAC1 presents a cholesterol binding pocket formed, in human isoform, by Ile^123^, Leu^144^, Tyr^146^, Ala^151^, and Val^171^ residues [23].

Mitochondrial porins form complexes with other proteins, such as the adenine nucleotide translocase (ANT), the translocator protein (TSPO), also known as the peripheral-type benzodiazepine receptor (PBR), mitochondrial HSP70, and several cytoskeletal proteins such as tubulin, actin, dynein light chain, and gelsolin [11].

The translocator protein interacts directly with all VDAC isoforms. In particular, interaction between TSPO and VDAC1 contributes to regulate the efficiency of mitochondrial quality control mechanisms and inhibits mitophagy, preventing ubiquitination of proteins through downregulation of the PINK1/Parkin pathway [24]. The GxxxG motif presents both in VDAC and in TSPO, and is necessary for this interaction [25]. Moreover, VDAC1 and TSPO, in association with StAR (steroidogenic acute regulatory protein), form the transduceosome, a multi-protein complex involved in cholesterol transport. In a former hypothesis, VDAC1 and TSPO in OMM, ANT in IMM, and Cyclophilin D in the mitochondrial matrix were candidates to constitute the permeability transition pore (PTP), a high conductance and non-specific pore that allows mitochondrial swelling and release of apoptogenic proteins. More recently, it was proposed that PTP could be formed by dimers of the ATP synthase complex [26].

Recent studies have focused attention on the role of VDAC proteins in mitochondrial dysfunction typical of many pathological conditions including stroke, cancer, mitochondrial encephalomyopathies, and aging, as well as neurodegenerative disorders [27,28].

Mass spectrometry analysis revealed the association between VDACs and the ubiquitin ligase Parkin. In presence of damaged mitochondria, as in Parkinson’s disease, Parkin is phosphorylated by PINK1 and consequently ubiquitinates proteins that reside on the OMM, targeting the mitochondria for degradation. Parkin is a cytosolic protein but translocates to the mitochondria to participate in mitochondrial quality control mechanisms. VDAC proteins represent a docking site of Parkin on defective mitochondria [29].

Moreover, VDAC1 represents the main docking site at the mitochondrial level for misfolded and aggregated proteins, a common feature of neurodegenerative disorders known as proteinopathies, such as Alzheimer’s disease (AD), Parkinson’s disease (PD), Creutzfeldt–Jacob disease (CJD), dementia with Lewy bodies (DLB), Huntington disease (HD), and amyotrophic lateral sclerosis (ALS) [30].

For example, in AD post-mortem brains, in neuroblastoma cells and in an AD mouse model, a direct association was demonstrated between VDAC1, specifically its N-terminal region, and hyper-phosphorylated Tau but also with amyloid beta (Aβ), both in its monomeric and oligomeric forms [31]. These interactions can have a dramatic effect on mitochondrial functions in AD neuron because they block the PTP formation, disrupt the transport of mitochondrial proteins and metabolites, and impair gating, conductance, and physiological interactome of VDACs [32].

In Parkinson’s disease, α-synuclein directly interacts with mitochondria, blocks VDAC1, and impairs metabolite fluxes leading, consequently, to an energetic crisis able to compromise cell viability [33].

In ALS, several SOD1 mutants are able to bind VDAC1 [34]. This interaction impairs ATP/ADP exchange, VDAC1 conductance and mitochondrial membrane potential. Recently, the competition between SOD1G93A and HK1 was demonstrated in binding VDAC1, in NSC34 motor-neuron cell lines [35].

In literature, the role of VDAC1 in neurodegeneration is rather well known; however, the involvement of the other two isoforms in these pathways remains poorly defined. This is likely associated with the relative abundance of VDAC1 compared to other isoforms which are more difficult to isolate in pure form.

Recent studies demonstrated that Cytoskeleton-associated protein 4 (CKAP4), a palmitoylated type II transmembrane protein localized to the endoplasmic reticulum (ER), regulates mitochondrial functions through an interaction with VDAC2 at ER-mitochondria contact sites [36].

VDAC2 binds inositol trisphosphate receptors (IP3R) and regulates the release of Ca^2+^ from the ER. In addition, several other interaction partners have been reported for VDAC2 isoform, which imply its effect in multiple cellular functions. Specifically, VDAC2 has been linked to many cellular proteins, including apoptotic factors as Bak and Bax, StAR, Metaxin2, eNOS (nitric oxide synthesize), GSK3β, tubulin, and Mcl1 [19]. In addition, VDAC2 and RACK1 (receptor of activated protein kinase C1) function as receptors for lymphocystis disease virus (LCDV) and for bursal disease virus in host cells [37].

VDAC2 together with VDAC3 binds Erastin, the activator of ferroptosis, a new pathway that regulates cell death characterized by the iron-dependent accumulation of lipid hydroperoxides. Interaction between VDAC2/3 and Erastin results in degradation of the channels following activation of ubiquitin protein ligase Nedd4 [38].

Finally, the VDAC3 isoform is associated with cytosolic proteins as tubulins and cytoskeletal proteins, stress sensors, chaperones, and proteasome components, redox-mediating enzymes such as protein disulfide isomerase [39].

## 2. MS-Based Techniques for Protein Analysis

The development in the late 1980s of the two “soft” desorption/ionization MS techniques electrospray ionization (ESI) and matrix-assisted laser desorption/ionization (MALDI) [40], which are able to convert large molecules, such as proteins, DNA, and carbohydrates, into gas-phase ions without affecting their integrity, has greatly broadened the applicability of MS to biology and revolutionized the analysis of biomolecules, making possible the high-throughput identification of thousands of proteins in only one experiment.

With respect to the ESI method, MALDI MS is commonly used for the characterization of relatively simple mixtures of peptides or proteins; it is quite resistant to interference with buffers commonly used in protein chemistry (e.g., phosphate, Tris, urea) and produces mass spectra that are simple to interpret because of the tendency of the method to generate predominantly singly charged molecular ions [41]. On the contrary, the most important feature of the ESI is the generation of multiply charged molecular ions. This feature allows the detection of proteins also using analyzers with limited mass range (e.g., quadrupoles and ion traps). ESI gained immediate popularity because it can be easily coupled on-line with high-performance separation techniques such as capillary electrophoresis and HPLC and currently represents the most used MS-based method in protein analysis [42,43].

In particular, chromatographic separation represents an essential step during protein analysis because highly complex samples, such as a total cellular protein fraction, may contain hundreds of components that must be, at least partially, separated before the MS analysis. Analogously, when a biological sample is digested, the complexity of the protein mixture increases because each protein component yields many peptides and a high-performing separation step is needed. A variety of liquid chromatographic (LC) separation methods, including reverse phase ultra-high-performance liquid chromatography (RP-UHPLC), strong cation exchange (SCX), affinity chromatography (AC), and size exclusion (SEC), have been developed [44]. Recently, UHPLC, operating with stationary phases consisting of small particles (size < 2 μm), was introduced for more efficient and faster peptide separation [44]. In order to improve the chromatographic resolving power, it is also possible to combine several separation techniques (i.e., multidimensional separation), in which each technique utilizes different physicochemical properties of molecules as a basis for separation (e.g., SCX joined with RP-HPLC) [45]. On the other hand, the development of high-resolution ion mobility mass spectrometry (IMMS), which allows ions to be separated on the basis of their size/charge ratios and their interactions with a buffer gas, adds a degree of orthogonality to MS, and it is now emerging as new powerful tool in the MS arena for the investigation of complex biological samples [46].

The implementation of separation techniques in miniaturized formats coupled on-line with high-performance mass spectrometers and the development of miniaturized ESI sprayers (nanoESI) have reduced the amount of analyte required for complete and routine sequence characterization below the attomole (10^−18^ mol) range. Moreover, the improved performance and versatility of the mass spectrometers nowadays allow to measure the molecular mass of proteins and peptides with high accuracy and to determine additional structural features, which include the primary structure or PTMs. In this respect, the mass analyzer plays a fundamental role in the mass spectrometer technology, together with its key parameters such as sensitivity, resolution, mass accuracy and the ability to generate MS/MS spectra.

Fundamentally, the mass analyzers currently used in protein studies are five: quadrupole (Q), ion trap (IT), time-of-flight (TOF), Fourier transform ion cyclotron resonance (FTICR), and Orbitrap. These mass analyzers differ considerably in design and performance, each with its own strength and weakness.

Ion trap, routinely coupled with ESI source, represents the unique analyzer which alone allows to perform both MS and tandem mass spectrometry (MS/MS) analysis. It is robust, sensitive, and moderately inexpensive, but shows a relatively low resolution and mass accuracy. TOF analyzer has a high sensitivity and high mass detection range that represent the main strengths of this type of analyzer in comparison with others [47]. This type of analyzer is dedicated for pulsed ionization methods, such as MALDI or secondary ion mass spectrometry (SIMS), whereas it cannot be directly interfaced with ESI sources. On the other hand, coupling quadrupole or ion trap analyzers with TOF (Q-q-TOF and LTQ-TOF, respectively) permits not only a direct interfacing with ESI but also allows MS/MS analysis.

Currently, FTICR MS [48] offers the highest resolution (more than 500,000 full width at half maximum, FWHM), resolving power, and accuracy greater than any other mass analyzer. Orbitrap [49], the unique mass analyzer developed during the last 20 years, uses the Fourier transform algorithm to obtain mass spectra with very high mass resolution (FWHM > 150,000 at *m*/*z* 600) and mass accuracy (<2 ppm) [50]. In particular, in these instruments, detection is based on the production of an induced alternating current (AC) with the same frequency as that of the oscillation of the ions inside the instruments. The complex signal produced must be mathematically processed by the Fourier transform algorithm.

Stand-alone FTICR and Orbitrap analyzers cannot perform ion fragmentation requiring additional fragmentation devices. The most common MS platforms equipped with these mass analyzers are the LTQ-FTICR and the LTQ-Orbitrap. In these systems, the LTQ mass analyzer is used for ion isolation, ion storage, and ion fragmentation in MS/MS experiments.

Although the resolution and accuracy of Orbitrap analyzer is not as high as in the case FTICR -MS spectrometer discussed above, Orbitrap offers higher scan rate, is more compact, less costly, easier to maintain, and does not require expensive cryogenic liquids. For these reasons, the LTQ-Orbitrap is becoming the universal solution when high resolution and mass accuracy are required in protein research.

The development of many early hybrid instruments was also motivated by the desire for MS/MS spectral acquisition, which represents one of the most important and effective analytical techniques among MS methodologies. MS/MS usually couples two MS steps and this enables to obtain information about the sequence of peptides.

During MS/MS procedure, a selected ion is isolated and fragmented. Fragmentation occurs along the polypeptide chain in a precisely defined manner. Each resulting fragment has its own mass-to-charge ratio (*m*/*z*) related to the *m*/*z* of the parent ion. By interpreting the MS/MS data, it is possible to reconstruct the sequence of peptides so obtaining structural information. Consequently, tandem MS plays a key role for protein or peptide sequencing and for the characterization of their PTMs. For this purpose, different fragmentation methods were introduced, such as collision-induced dissociation (CID), electron-capture dissociation (ECD), electron-transfer dissociation (ETD), and higher-energy C-trap dissociation (HCD). CID is the most widely applied fragmentation method for MS/MS in proteomics, mainly resulting in cleavage of amide bonds [51,52]. CID is more effective for small, low-charged peptides, whereas it is not suitable for fragmentation of intact proteins and peptides with labile PTMs. In addition, in spite of its prevalence, CID provides limited sequence coverage when applied to peptide sequences carrying many basic residues. The recent development of the new fragmentation techniques of ECD, ETD, and HCD has greatly enlarged the capabilities of tandem MS strategies [53,54,55], providing data highly complementary to the conventional CID, better sequence coverage for small-sized to medium-sized peptides, and more comprehensive characterization of PTMs. Therefore, taking into account the complementary fragmentation information that can be obtained by these methods, the use of CID, ETD, or HCD alone, in alternating acquisition or by a decision-tree regulated combination, can provide the best performance for the analysis of distinct peptide populations [56].

Because a detailed description of the capabilities of the different mass analyzers, hybrid instruments, and fragmentation methods is out of the purpose of the present chapter, the reader is referred to a recently published review [57].

## 3. Proteomics of VDAC Isoforms

### 3.1. Sample Preparation

Sample preparation has a profound effect on the final results of a proteomic workflow. Protein extraction methods and protein separation techniques should provide an unbiased and reliable map representative of all proteins present in a specific sample. The different extraction and fractionation approaches are based on proteins physicochemical and structural characteristics, such as molecular weight, solubility, hydrophobicity, and isoelectric point. A specific protocol has to be optimized for each particular sample, to maximize protein recovery and minimize the possible proteolysis and amino acid modifications. For these reasons, there is no universal extraction protocol and not a unique buffer composition. Regarding the extraction method, the different strategies available need to be compatible with both the amount of the processed material and the subsequent analytical approach (i.e., separation or MS).

The structural characterization of VDACs presents challenging issues due to their very high hydrophobicity, low solubility, and the impossibility to separate them from other mitochondrial proteins of similar hydrophobicity and to easily isolate each single isoform. In fact, isolation of VDACs has been possible exclusively for plant VDAC isoforms by chromatofocusing, thanks to the absence of phosphorylation sites in their structure [58]. Consequently, it is necessary to analyze them as components of a relatively complex mixture.

A bottom-up proteomic approach was used to investigate the VDAC3 from rat liver mitochondria (rVDAC3) [59]. According with a standard procedure [60], mitochondria were extracted and lysed with a buffer containing 3% Triton X-100 at pH 7.0. The VDAC proteins were partially purified by hydroxyapatite (HTP) chromatography, which allows to obtain a VDACs enriched fraction which comprises also other mitochondria hydrophobic proteins. After precipitation with cold acetone, the protein pellet was solubilized in SDS buffer and loaded on a 17% polyacrylamide gel (1D-SDS-PAGE). The bands in the range 30–35 kDa were manually excised from the gel, cut in small pieces, and subjected to reduction with DTT and alkylation by addition of IAA. Finally, the reduced and carboxyamidomethylated proteins were in gel-digested using trypsin and chymotrypsin, and the resulting peptide mixtures were analyzed by nUHPLC/HRMS [59]. MS data showed that rVDAC3 was found in the whole range 30–35 kDa, together with other proteins, mainly VDAC1, VDAC2, and several other mitochondrial proteins. The reason for VDAC3 electrophoretic heterogeneity probably stems from (i) the different pattern of cysteine oxidations that can modify the protein mobility; (ii) the different amount and quality of cysteine oxidations in various molecules (“redox isomers”).

The gel-digestion procedure shows some disadvantages: (i) larger peptides can get trapped between the gel meshes and lost during the extraction phase of the peptides from the gel; (ii) the electrophoretic procedure itself could damage the samples and alter the redox state of the sulfur amino acids (due to possible over heating generated by the applied voltage and to the presence of residual quantities of the catalysts used for the polyacrylamide polymerization). Furthermore, electrophoresis requires a greater amount of sample and the utilization of dyes and detergents: These last molecules could interfere with subsequent MS analyses because these compounds are difficult to eliminate from the sample.

2-DE could potentially represent a useful alternative to 1-DE to improve the separation of VDAC isoforms, but its utilization presents other problems. Actually, this kind of proteins has been under-represented in 2-DE gels due to difficulties in extracting and solubilizing them in the isoelectric focusing sample buffer. In fact, the most effective solubilizing agent for highly hydrophobic membrane proteins is SDS, but this detergent is incompatible with 2-DE. In addition to the difficulties in entering IPG (immobilized pH gradient) gels, membrane proteins tend to precipitate at their isoelectric point during IEF. Furthermore, their tendency to absorb the IPG matrix prevents their migration into the SDS-PAGE gel.

An improvement in the rVDAC3 mass spectrometric analysis was obtained following the introduction of a gel-free shotgun proteomic approach [59]. According to this procedure, to avoid any possible artefact due to air exposure and manipulations, reduction/alkylation was carried out before VDACs purification from the mitochondria. Afterwards, all the proteins present in the HTP eluate, without previous electrophoretic separation, were purified from non-protein contaminating molecules with the PlusOne 2-D Clean-Up kit, and the desalted protein pellet was then re-dissolved in ammonium bicarbonate containing RapiGest SF to improve the solubility. In fact, this surfactant makes the proteins more susceptible to enzymatic cleavage without modifying the sample or inhibiting endoprotease activity. Furthermore, the RapiGest SF is compatible with enzymes such as trypsin or chymotrypsin and does not influence subsequent MS analysis because it can be easily removed in acidic conditions.

Separate aliquots of reduced and alkylated proteins were then subjected to digestion with modified porcine trypsin and chymotrypsin. In this experiment, every protein in the HTP eluate was digested, producing a very complex peptide mixture, which was finally analyzed by nUHPLC/HR nESI-MS/MS.

The new “in solution-digestion” protocol associated with nUHPLC/HR ESI-MS/MS allowed to extend the coverage of the rat and human VDACs sequences with respect to that obtained with the previous procedure [61], so that it was possible to completely cover the rat and human VDAC1 sequences and almost completely the rat and humanVDAC2 and VDAC3 sequences [22,59,62]. It should be noted that the short regions not identified in VDAC2 and VDAC3 correspond to small tryptic or chymotryptic peptides or even to single amino acids, which cannot be detected in LC/MS analysis.

Moreover, by means of this new procedure a detailed characterization of PTMs of the three VDACs was obtained (see next paragraphs).

### 3.2. Mass Spectrometry Analysis of Post-Translational Modifications

The mammalian proteome is vastly more complex than the related genome. The reasons for this difference reside both in the molecular mechanisms that allow a single gene to encode for multiple proteins (genomic recombination, transcription initiation at alternative promoters, differential transcription termination, and alternative splicing of the transcript) and in the post-translational modifications (PTMs) which represent a wide range of chemical changes that proteins can undergo after synthesis. They include the specific cleavage of protein precursors, the covalent addition or removal of low-molecular groups (i.e., acetylation, glycosylation, hydroxylation, phosphorylation, ubiquitination) and the formation of disulfide bonds or other redox modifications [63,64,65].

PTMs play crucial roles in cell biology since they can change protein physical or chemical properties, activity, localization, and/or stability. Traditionally, PTMs have been identified by Edman degradation, amino acid analysis, isotopic labeling, or immunochemistry. Within recent years, MS has proven to be extremely useful in PTM discovery. Post-translationally modified amino acids always have a different molecular mass than the original, unmodified residues and this mass increment or deficit is usually the basis for the detection and characterization of PTM by MS (commonly by LC-ESI-MS/MS).

MS has several advantages for characterization of PTMs, including (i) very high sensitivity; (ii) discovery of novel PTMs; (iii) ability to identify PTMs and the modified sites, even in complex protein mixtures; and (iv) ability to quantify the relative changes in PTM occupancy at distinct sites. None of the other techniques provide all these features, so the greater majority of the known PTMs have been described by MS [66].

**Figure 1:**
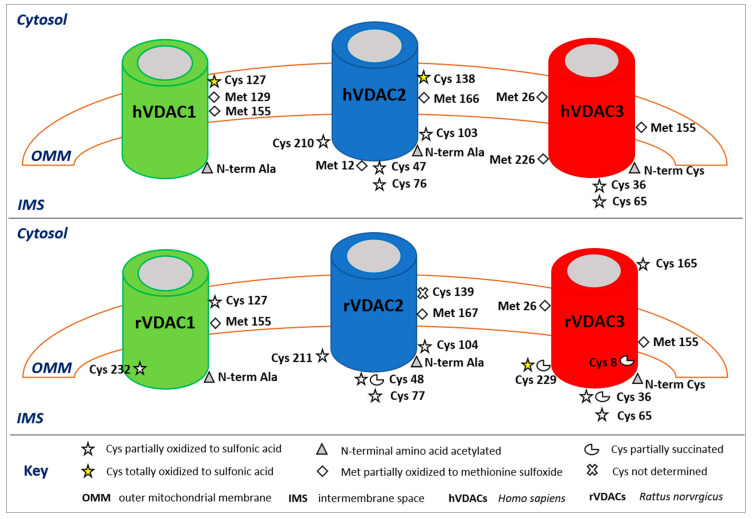
Post-translational modifications of human (upper panel) and rattus (lower panel) VDAC isoforms. The image shows only the modified amino acids and their positions with respect to the cytosol, the outer mitochondrial membrane (OMM), and the intermembrane space (IMS). In the rVDAC1 Cys^232^ faces the aqueous inside of the pore; in the rVDAC3 Cys^8^ is located inside of the pore.

To improve sensibility, several methods have been developed to enrich the samples in proteins or peptides with specific PTMs prior to MS/MS analysis, such as anti-pY antibodies, IMAC (immobilized metal affinity chromatography) and TiO_2_ for phosphorylation [67,68], affinity capture with lectins for glycosylated proteins [69], and resin coupled with anti-acetyl-lysine for acetylated proteins [70]. Although, as previously described, isolation of single isoforms of VDACs cannot be obtained, application of combined HPLC and high-resolution ESI-MSMS analysis has resulted in the identification of several PTM in these proteins. In the following, a summary of the MS-based PTMs characterized in VDACs is reported and the respective biological significance discussed. These results are resumed in Table 1 and Figure 1.

#### 3.2.1. Deletion of N-Terminal Methionine and Acetylation of Lysines

In all rat and human VDACs the HRMS data confirmed that the N-terminal Met, reported in the SwissProt database sequence, is missing in the mature proteins and that the N-terminal amino acid is always acetylated [22,59,62]. Although N-terminal acetylation is widespread in eukaryotes, the biological relevance of this modification is only known for a few substrates [71,72]. Unlike ε-lysine modification, N-terminal acetylation, catalyzed by N-terminal acetyltransferases, is irreversible and occurs co-translationally [73,74]. It has been suggested that co-translational N-acetylation modifies protein–protein interaction, affects accumulation of the mature protein(s) in target organelles [75], confers metabolic stability to the protein by providing general protection from peptidases and against irreversible oxidation, and seems to act as a degradation signal in the Ac/N–degron pathway [76]. Consequently, any dysregulation of N-terminal acetylation, leads to serious pathological conditions including neurodegenerative diseases, cancers, hypertension, and X-linked genetic disorders [77,78,79].

All data regarding the acetylation of the three different VDAC isoforms were obtained from proteomic studies of acetyl-lysine enriched fractions.

In liver of fed and starved mice, lysines 33, 41, 74, and 234 of VDAC1 were detected acetylated by immunoprecipitation enrichment of acetylated peptides with an anti-acetylated lysine antibody and nanoHPLC MS/MS in an LTQ mass spectrometer [70]. Analogously, using mass spectrometry and label-free quantitation, Schwer et al. found that, in mouse liver tryptic digest immunopurified with acetyl-lysine affinity matrix, lysines 41, 122, and 132 in VDAC1 were found acetylated during calorie restriction [80]. However, no significant quantitative difference in VDAC1 acetylation was highlighted between fed and starved and fed and calorie restricted animals, respectively. In addition to the above-mentioned sites, lysine 237 was also acetylated in mouse liver and heartVDAC1 analyzed with a built-in-house Velos-FT mass spectrometer [81].

Acetylation of lysines 32 and 75 [70] and 121 [80] was observed in mouse liver VDAC2. Again, no differences in acetylation between fed and starved and fed and calorie-restricted mice were revealed.

In contrast to VDAC1 and VDAC2, VDAC3 showed acetylation of lysines 20, 61, and 226 only in livers of starved but not of fed mice [70]. In addition, lysines 63 and 109 were identified acetylated in mouse liver, with no differences between fed and calorie-restricted animals [80]. However, none of the above studies addressed the potential physiological effect of VDAC acetylation.

In human liver mitochondria, VDAC1 and VDAC3 were found acetylated in position 28 after purification with an antibody to acetyl lysine and analysis by tandem liquid chromatography–tandem mass spectrometry (LC/LC-MS/MS) [82]. The amino acid sequence of the acetylated dodecapeptide in human VDAC1 and 3 (GYGFGLIK*LDLK and GYGFGMVK*IDLK, respectively) differs in three amino acid residues.

#### 3.2.2. Oxidation States of Methionines

Even in mild conditions, methionine residues are highly susceptible to oxidation mediated by reactive oxygen (ROS) and nitrogen species (NOS) to generate in vivo Met sulfoxide (MetO), which can be further oxidized to methionine sulfone (MetO_2_) under stronger oxidizing conditions [83]. MetO exists as a mixture of S and R diastereomers and its production is tightly regulated in vivo by ubiquitous sulfoxide reductases, which catalyze the thioredoxin-dependent reduction of MetO into methionine [84]. Conversely, MetO_2_ cannot be reduced in vivo.

Cyclic Met oxidation/MetO reduction leads to consumption of ROS and thereby acts as a scavenging system to protect proteins from oxidative damage [85,86]. Moreover, MetO formation seems to have little or no effect on protein susceptibility to proteolytic degradation [87,88]. Numerous studies reported that MetO levels in proteins increase during aging and in certain disease, in particular in the neurodegenerative ones [89]. Evidence for an additional role of certain methionines as oxidation sensors in the redox regulation of enzyme activity is accumulating [90,91,92].

In rat and human VDAC isoforms, methionines oxidized to sulfoxide were detected by nUHPLC/HRnESI-MS/MS [22,59,62].

Considering the VDAC1 sequence, one methionine residue (Met^155^) is conserved in rat and human. The mass spectral data indicated that among the peptides identified, besides the fragments containing Met^155^ in the normal form, peptides with this residue in the form of methionine sulfoxide (MetO) were also identified [93].

Although from these data a precise determination of the ratio between Met and Met sulfoxide could not be obtained, a rough estimation of their relative abundance was derived from the comparison of the absolute intensity of the multiply charged molecular ions of the respective peptides. These calculations demonstrated that in rat VDAC1 (rVDAC1) the conserved Met^155^ is oxidized to MetO in a remarkable higher amount (Ox/Red ratio 65:1) than in the human VDAC1 (hVDAC1) (Ox/Red ratio 0.8:1), while the Ox/Red ratio for the Met^129^, which is present only in hVDAC1, resulted 1.5:1.

Analogously, VDAC2 sequence contains one internal conserved methionine, Met^167^ in rat and Met^166^ in human, respectively. Both the amino acid residues are partially oxidized to MetO in approximatively equal amount (Ox/Red ratio 3.2:1 in rVDAC2, and 1.4:1 in hVDAC2). On the contrary, the other Met^12^ in hVDAC2 is mainly in the oxidized form (Ox/Red ratio about 10:1).

Finally, in the hVDAC3 the Ox/Red ratio of the three methionines 26, 155, and 226 was about 0.1:1, 1:1, and 3:1, respectively. In particular, Met^26^ showed an oxidation state comparable to that of the analogous rVDAC3 methionine (Ox/Red ratio about 0.6:1), whereas the oxidation rate of Met^155^, the latter conserved methionine, was not determined in the rat isoform.

Literature has highlighted a key role of VDACs in mitochondrial dysfunction typical of many neurodegenerative disorders. In particular, the principal isoform VDAC1 represents the main mitochondrial docking site of many misfolded proteins, such as amyloid-β and Tau in Alzheimer’s disease, α-synuclein in Parkinson’s disease, and several SOD1 mutants in ALS. The interaction of misfolded proteins with VDAC1 has a strong impact on both cellular bioenergetics and apoptosis’ pathways alteration. Therefore, VDACs represent a promising therapeutic target in neurodegenerative diseases [28].

Very recently, by HRMS/MS analysis, possible signs of oxidative damage in VDAC1 from the NSC34-SOD1G93A cell line, a suitable ALS motor neuron cell model line, were investigated [94].

Interestingly, a higher amount of methionine sulfoxide and sulfone was observed for Met^155^ in the mutated line (MetO/Met ratio 61:1 and MetO_2_/Met 4.7:1, respectively) in comparison with NSC34 and NSC34-SOD1WT cell lines (MetO/Met ratio 3–5:1 and MetO_2_/Met0.1:1, respectively).

#### 3.2.3. Oxidation States of Cysteines

Because of their redox-reactive thiol (–SH) side chain, cysteine residues are easily subjected to various Ox-PTMs including S-nitrosylation (or S-nitrosation, SNO), sulfhydration (SSH), S-acylation, S-glutathionylation, sulfenylation (SOH), and oxidation to sulfinic acid (SO_2_H) and sulfonic acid (SO_3_H) [65]. Except for sulfonic acid, all the reported Ox-PTMs are readily reversible and ruled by specific enzymatic activities. Moreover, cysteine oxidation has a structural relevance for proteins, since it can support the disulfide bridge formation (RS-SR), a physiological protein cross-linking, which is an essential PTM involved in protein folding and in the stabilization of tertiary and quaternary structure. Disulfide formation depends on the spatial proximity between two cysteines and can also occur through a reaction with sulfenic acid in the presence of high concentrations of ROS. They indeed convert SOH groups into thiol radicals (RS) which, in turn, react with other thiolates to form a disulfide bond [65].

In a recent paper, mitochondrial enrichment and subsequent cysteine-targeted MS analysis allowed to identify ~1500 reactive cysteine residues on ~450 mitochondrial proteins in HEK293T cells, and additionally, 22 highly S-nitrosoglutathione (GSNO)-sensitive cysteines also in VDAC proteins [95].

Through UHPLC/HR nESI-MS/MS analysis, cysteine PTMs were characterized in detail, and it was discovered that such cysteines can be subject to different oxidization degrees, ranging from the disulfide bridge to the most oxidized, the sulfonic acid one, an irreversible and permanent protein modification, and even harmful in the cell. In fact, as mentioned above, there are no known enzymes that can reverse this form back to any of the lower sulfur oxidative states (sulfenic and sulfinic forms) [96].

Interestingly, the insertion of a strong negative charges by –SO_3_H formation can modify the protein conformation by electric repulsions inside the chain or toward phospholipids. Some authors suggested that these conformational changes can initiate protein incorporation into mitochondria-derived vesicles (MDVs), later targeted to lysosomes. MDVs, whose production is induced by mitochondrial stress [97], contain numerous oxidized proteins derived mainly from the OMM.

Recent findings revealed a precise and evolution conserved pattern in oxidative status among human and rat VDACs [22,59,62].

VDAC1 presents only two cysteines in position 127 and 232, located far from each other. In rVDAC1 both these residues are moderately oxidized, with a ratio Ox/Red in the range of 0.1–0.26 [62]. Instead, in hVDAC1, Cys^127^ (in the β-strand 8), which protrudes in the hydrophobic, phospholipidic milieu, is totally trioxidized as –SO_3_H, while Cys^232^ (in β-strand 16) facing the water-accessible side of the channel [5,6,7], is exclusively in the reduced and carboxyamidomethylated form [22].

VDAC2 is the longer isoform, with an N-terminal extension of 11 amino acids in mammals and is the richest in cysteines: 11 and 9 in rat and human, respectively. This difference is due to the presence of two additional Cys residues in the N-terminal sequence of the rat isoform.

In hVDAC2 Cys^8^, Cys^13^, and Cys^133^ were exclusively in the reduced form; these residues are exposed to the IMS and correspond to Cys^9^, Cys^14^, and Cys^134^ in rVDAC2, which have also been found to be reduced or not determined (Cys^134^).

Cysteines in position 47, 76, 103, and 210, all in IMS loops, were instead identified as partially oxidized to –SO_3_H. Among them, Cys^76^ was detected in the form of sulfonic acid with an Ox/Red ratio of about 0.1:1, similarly to the homologue Cys^77^ in rVDAC2. Cysteines 47, 103, and 210 are found partially trioxidized but with a lower Ox/Red ratio (about 0.1:1–0.01:1), reproducing the trend observed for the homologous cysteines 48, 104, and 211 of rVDAC2.

Cys^227^, which is instead the only cysteine predicted to be in a hydrophilic turn exposed to the cytosol, resulted exclusively reduced, analogously to the Cys^228^ in rVDAC2, whose trioxidized form was detected in very low amount (rough ratio of 1:100 oxidized/reduced). It is tempting to speculate that it can be involved in some kind of docking function of this protein.

In rVDAC2, the additional Cys^4^ and Cys^5^ in the N-terminal moiety are also reduced; thus, VDAC2 has a cluster of cysteines oriented towards the IMS that are available to reversible oxidation.

Finally, hVDAC2 Cys^138^, situated in a position similar to that of Cys^127^ in hVDAC1, and therefore embedded in the hydrophobic environment of the OMM, was found fully trioxidized to sulfonic acid as the homologous residue in hVDAC1.

It is important to underline that the preferred redox state of cysteines in general is conserved between rat and human VDAC3 [22,59]. Accordingly, Cys^36^ and Cys^65^ were detected in a reduced form and trioxidized to sulfonic acid in a similar extent in both organisms. Cys^2^ and Cys^122^, which are also facing the IMS, were identified entirely reduced, as Cys^8^ which is inside the channel. In rat, the oxidation state of Cys^122^ remained undetermined because peptides containing this residue were not detectable. The only exception was Cys^229^ that in hVDAC3 is completely reduced, whereas in the rat isoform was totally trioxidized.

Overoxidation can be required to target a protein to degradation. In particular, in the so called “N-end rule pathway”, the presence of an oxidized N-terminal cysteine in certain mammalian proteins is required for arginylation by ATE1 R-transferases and subsequent ubiquitin-dependent degradation [98]. In VDAC3, Cys^2^ becomes the N-terminal end after the elimination of Met^1^ during the protein maturation [22,59]. Because the oxidized form of Cys^2^ was never detected, this can mean that the potentially Cys^2^-oxidized, if existing, is a marker used to remove the protein from the membrane for degradation.

On the contrary, the groups of Cys residues always in reduced form in all the three VDAC isoforms could have another function: The persistence of their reduction, together with the exposition to the IMS, an oxidative environment, prompts to speculate their involvement in disulfide bridge(s) formation, with conformational consequence for the pore.

In conclusion, it is important to underline that most of the HRMS data obtained for the oxidation states of cysteines in human VDACs are the same as those obtained for the rat ones, thus demonstrating that they do not depend on the type of organism, at least in mammals, but reflect a physiological condition.

Furthermore, the power of the deep mass spectrometric analysis reported us a list of other proteins present in the HTP eluate from Triton X-100 solubilized rat liver mitochondria. Among them, no evidence of over-oxidized cysteines was discovered, demonstrating that the overoxidation is a peculiarity of the VDAC isoforms [62]. Another validation of these data is that the application of the same methodologies to different kind of sources, living tissue from organism (rat liver), or cultured cells (human HAP cell) resulted in remarkably similar results.

#### 3.2.4. Phosphorylation

Phosphorylation of VDACs was reported in pathological conditions and is associated with degradation and pro-apoptotic pathways [14,99]. Indeed, some results suggest that VDAC1 phosphorylation is involved in the genesis of apoptosis in brain of Alzheimer disease (AD) and Down syndrome (DS) patients [100]. In this work, the authors described protein levels of VDACs in individual post-mortem brain regions of patients with DS and AD using two-dimensional electrophoresis (2-DE) and MALDI TOF-MS. VDAC1 and VDAC2 were unambiguously identified and quantified, but VDAC3 was not found. It was noted an increase in the content of three VDAC1 isoforms, which differed in their isoelectric points, probably caused by post-translational modifications as, e.g., phosphorylation. However, no information regarding the type and site of phosphorylation was presented.

Through proteomic analysis followed by Western blotting and immunohistochemical techniques, it was demonstrated that VDAC1 is overexpressed in the hippocampus from amyloidogenic AD transgenic mice models [101]. VDAC1 was also overexpressed in postmortem brain tissue from AD patients at an advanced stage of the disease. Interestingly, amyloid-β (Aβ) soluble oligomers were able to induce upregulation of VDAC1 in a human neuroblastoma cell line, further supporting a correlation between Aβ levels and VDAC1 expression. In hippocampal extracts from transgenic mice, a significant increase was observed in the levels of VDAC1 phosphorylated at an epitope susceptible to phosphorylation by glycogen synthase kinase-3β [101].

Protein phosphorylation is catalyzed by a family of protein kinases, with serine, threonine, and tyrosine side chains being the most frequently modified [102]. The reverse reaction is catalyzed by specific protein phosphatases, i.e., phosphoserine/phosphothreonine-specific and phosphotyrosine-specific families of phosphatases [103].

To study the PTMs of rVDACs, a method based on enrichment of the proteins by harvesting high-purity outer membrane from rat liver mitochondria, followed by isolation of the proteins of interest by semipreparative SD-PAGE/electroelution was developed [104]. Following this approach, all three isoforms were found phosphorylated at one or more sites, in particular, VDAC1 at serine 12 and 136, corresponding to CaM-II/GSK3 and PKC consensus sites, respectively. Employing an improved IMAC method to examine mouse liver mitochondrial phosphoproteome, it was documented also the phosphorylation of serine 117 [105]. By a shotgun approach, phosphorylation of serine 101, 102, and 104, and of threonine 107 in HeLa cells were determined [106] as well as of tyrosine residues 80 and 208 in mouse brain [107].

VDAC2 phosphorylation of serine 115 and threonine 118 was demonstrated in HeLa cells [106]; in rat liver mitochondria Deng et al. reported that VDAC2 is phosphorylated at threonine 109 [108], whereas Distler et al. found phosphorylation at tyrosine 237 [104]. Subsequently, in mouse brain Ballif et al. documented tyrosine phosphorylation of residue 207 [107].

Finally, in VDAC3 from rat liver mitochondria, phosphorylation of serine 241 and threonine 33 was shown [104]. These sites correspond to PKA and PKC consensus sites. In mouse brain, VDAC3 is also tyrosine-phosphorylated at residue 49 [107].

**Figure 2:**
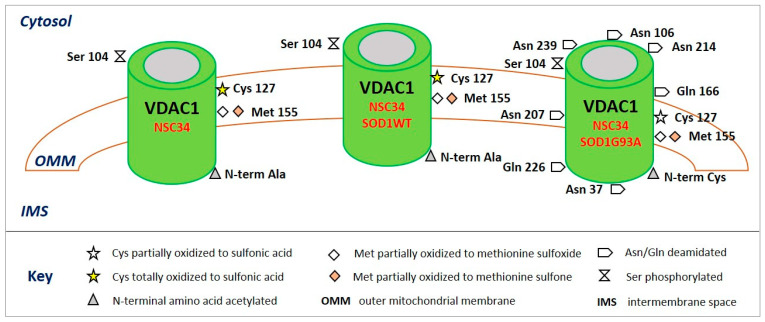
Post-translational modifications of VDAC1 from NSC34 (left), NSC34-SOD1WT (middle), and NSC34-SOD1G93A (right) cell lines. The image shows only the modified amino acids and their positions with respect to the cytosol, the outer mitochondrial membrane (OMM), and the intermembrane space (IMS).

Again, the phosphorylation of Ser^104^ in VDAC1 was evidenced in the three cellular lines (NSC34, NSC34-SOD1WT, and NSC34-SOD1G93A) recently investigated by nUHPLC/HR ESI-MS/MS, even though in low amounts (Table 2 and Figure 2) [94].

#### 3.2.5. Succination, Deamidation, Ubiquitin, Ubiquitination, and Presence of Selenocysteines

Elevated fumarate concentrations as a result of Krebs cycle inhibition lead to increases in protein succination, an irreversible post-translational modification that occurs when fumarate reacts with cysteine residues to generate S-(2-succino)cysteine (2SC). It has been verified in some mitochondrial diseases [109], cancer (with fumarate hydratase (FH) deficiency) [110], and diabetes [111].

Using the Ndufs4 knockout (Ndufs4 KO) mouse, a model of Leigh syndrome, it was demonstrated that protein succination increased in the brainstem (BS). 2D-SDS-PAGE followed by immunoblotting for succinated proteins and MS/MS analysis of BS proteins allowed to identify VDAC1 and 2 as specific targets of succination: in particular, Cys^77^ and Cys^48^ were identified as endogenous sites of succination in VDAC2. Given the important role of the VDACs in the exchange of ADP/ATP between the cytosol and the mitochondria, and the already decreased capacity for ATP synthesis in the Ndufs4 KO mice, the authors proposed that the increased protein succination observed in the BS of these animals would further decrease the already compromised mitochondrial function [109].

Identification of succinated cysteines in rVDAC2, but not in rVDAC1, was also reported [62]. In rVDAC2 only Cys^48^ was succinated in very low amount with a ratio succinated/normal about 0.04.

Selenocysteines were not observed in any of the rVDAC isoforms. In the nUHPLC/HRESI-MS/MS analysis of the PTMs of VDAC1 in ALS-SOD1 model cells [94], succinated cysteines were not found, as well as no evidence of ubiquitin and ubiquitination.

Notably, in the same work, the deamidation of five specific asparagine (Asn^37^, Asn^106^, Asn^207^, Asn^214^, and Asn^239^) and two glutamine (Gln^166^ and Gln^226^) residues was revealed exclusively in VDAC1 purified from NSC34-SOD1G93A cells but not in NSC34-SOD1WT or NSC34 cells. Deamidation is a non-inheritable PTM that introduces negative charges by removing amino groups from asparagine and, with a much lower frequency and rate, glutamine [112,113].

Because deamidation alters both peptide and protein structure and charge, it is suspected to contribute to the aging proteome in stability and to protein folding disorders such as those observed in Alzheimer’s, Parkinson’s, and many other degenerative diseases [114,115,116,117,118]. In particular, it has been found that deamidation is one of the most abundant modifications in long-lived proteins and can serve as disease biomarkers. Moreover, abnormal level of deamidation has also been associated with disease-related proteins in type 2 diabetes and cancers, and therefore, in-depth characterization of deamidation is of great biological significance [119].

The deamidation level of the Asn^207^ residue was much higher than for any other residue converted to aspartate. This residue is indeed located in the center of the β-strand 14 and is preceded and followed by non-bulky amino acid residues. As a consequence, the amido group of Asn^207^ can be particularly sensitive to ROS at the IMM, which are strongly increased in ALS, as well as being affected by the aggressive action of peroxidized lipids in the OMM.

Interestingly, the different charge distribution due to deamidation must have an impact on the physiological interactivity of VDAC1 and consequently on the functionality of the protein itself and of the whole mitochondrion.

In conclusion, these post-translational changes in VDAC1 may be involved in its specific interaction with ALS-related SOD1 mutants and it is reasonable to hypothesize a role of the modified isoform in the disease and as a biomarker of irreparably damaged mitochondria, and at the same time for the early diagnosis of this pathology.

## 4. Conclusions

The remarkable difficulties in the analysis of VDAC are related to their very high hydrophobicity, low solubility and the impossibility to separate them from each other. Currently available combination of nUHPLC and HRMS provides a powerful and indispensable tool for the detailed structural characterization of a single protein in a complex mixture without previous isolation, including the fine characterization of PTMs. Future advances in technology are expected in the next years. New instrumentation able to performs faster scanning speeds and higher mass accuracy will allow for more peptide identifications and ultimately higher proteome coverage which will facilitate the analysis of membrane proteins.

Presently, the association of an originally developed “in-solution” protocol with nUHPLC/HRMS for the analysis of an enriched VDACs fraction has permitted a fine determination of a number of PTMs in these proteins. As a result, the use of specific PTMs as possible biomarkers for an early diagnosis of degenerative diseases can be envisaged.

The assignment of a functional role to these modifications of VDACs will be a further step towards the full understanding of the roles of these proteins in the cell, leading to an even more detailed knowledge of the molecular basis of the degenerative disorders which are still poorly understood.

## Figures and Tables

**Table 1:** Post-translational modifications in VDAC isoforms obtained using mass spectrometry. PTM type, mass shift (Da), source of the sample, modified residue, MS method and relative reference are reported. Studies are described by listing first author + year.

ISOFORM	PTM Type	ΔMass (Da)	Source	Residue	Method	Study
VDAC1	Protein N-terminal acetylation	42.0106	Rat liver	Ala 2	nUHPLC/high resolution nESI-MS/MS in a Q-QT-qIT MS	Saletti et al., 2018
			HAP1 cells	Ala 2		Pittalà et al., 2020
	Acetylation	42.0106	Mouse liver	Lys 33, 41, 74, 234	nHPLC MS/MS in an LTQ MS	Kim et al., 2006
				Lys 41, 122, 132	nHPLC MS/MS in an LTQ 2D ion-trap MS	Schwer et al., 2009
			Mouse liver and heart	Lys 237	UPLC Velos-FT MS	Yang et al., 2011
			Human liver	Lys 28	LC/LC-MS/MS in an FTICR/MS	Zhao et al., 2010
	Oxidation	15.9949	Rat liver	Met 155	LC/LC-MS/MS in an FTICR/MS	Guan et al., 2003
					nUHPLC/high resolution nESI-MS/MS in a Q-QT-qIT MS	Saletti et al., 2018
			HAP1 cells	Met 129, 155		Pittalà et al., 2020
	Trioxidation	47.9847	Rat liver	Cys 127, 232		Saletti et al., 2018
			HAP1 cells	Cys 127		Pittalà et al., 2020
	Phosphorylation	79.9663	Rat liver	Ser 12, 136	HPLC MS/MS in an LTQ MS	Distler et al., 2007
			Mouse liver	Ser 117	nHPLC MS/MS in an LTQ MS	Lee et al., 2007
			HeLa cells	Ser 101, 102, 104, Thr 107	nHPLC MS/MS in an LTQ-Orbitrap MS	Olsen et al., 2006
			Mouse brain	Tyr 80, 208	LC-MS/MS in an LTQ FT MS	Ballif et al., 2008
VDAC2	Protein N-terminal acetylation	42.0106	Rat liver	Ala 2	nUHPLC/high resolution nESI-MS/MS in a Q-QT-qIT MS	Saletti et al., 2018
			HAP1 cells	Ala 2		Pittalà et al., 2020
	Acetylation	42.0106	Mouse liver	Lys 32, 75	nHPLC MS/MS in an LTQ MS	Kim et al., 2006
				Lys 121	nHPLC MS/MS in an LTQ 2D ion-trap MS	Schwer et al., 2009
	Oxidation	15.9949	Rat liver	Met 167	nUHPLC/high resolution nESI-MS/MS in a Q-QT-qIT MS	Saletti et al., 2018
			HAP1 cells	Met 12, 166		Pittalà et al., 2020
	Trioxidation	47.9847	Rat liver	Cys 48, 77, 104, 211		Saletti et al., 2018
			HAP1 cells	Cys 47, 76, 103, 138, 210		Pittalà et al., 2020
	Succination	116.0110	Mouse brain	Cys 48, 77	LC-nESI-MS/MS in an LTQ-Orbitrap MS	Piroli et al., 2016
			Rat liver	Cys 48	nUHPLC/high resolution nESI-MS/MS in a Q-QT-qIT MS	Saletti et al., 2018
	Phosphorylation	79.9663	HeLa cells	Ser 115, Thr 118	nHPLC MS/MS in an LTQ-Orbitrap MS	Olsen et al., 2006
			Rat liver	Thr 109	SCX-RP-MS/MS in an LTQ-Orbitrap MS	Deng et al., 2010
			Rat liver	Tyr 237	HPLC MS/MS in an LTQ MS	Distler et al., 2007
			Mouse brain	Tyr 207	LC-MS/MS in an LTQ FT MS	Ballif et al., 2008
VDAC3	Protein N-terminal acetylation	42.0106	Rat liver	Cys 2	nUHPLC/high resolution nESI-MS/MS in a Q-QT-qIT MS	Saletti et al., 2016
			HAP1 cells	Cys 2		Pittalà et al., 2020
	Acetylation	42.0106	Mouse liver	Lys 20, 61, 226	nHPLC MS/MS in an LTQ MS	Kim et al., 2006
				Lys 63, 109	nHPLC MS/MS in an LTQ 2D ion-trap MS	Schwer et al., 2009
			Human liver	Lys 28	LC/LC-MS/MS in an FTICR-MS	Zhao et al., 2010
	Oxidation	15.9949	Rat liver	Met 26, 155	nUHPLC/high resolution nESI-MS/MS in a Q-QT-qIT MS	Saletti et al., 2016
			HAP1 cells	Met 26, 155, 226		Pittalà et al., 2020
	Trioxidation	47.9847	Rat liver	Cys 36, 65, 165, 229		Saletti et al., 2016
			HAP1 cells	Cys 36, 65		Pittalà et al., 2020
	Succination	116.0110	Rat liver	Cys 8, 36, 229		Saletti et al., 2018
	Phosphorylation	79.9663	Rat liver	Ser 241, Thr 33	HPLC MS/MS in an LTQ MS	Distler et al., 2007
			Mouse brain	Tyr 49	LC-MS/MS in an LTQ FT MS	Ballif et al., 2008

**Table 2:** Post-translational modifications in VDAC1 from NSC34, NSC34-SOD1WT, and NSC34-SOD1G93A cell lines obtained using mass spectrometry. PTM type, mass shift (Da), cell line, residue, and modified/normal ratio are reported.

PTM Type	ΔMass (Da)	Cell Line	Residue	Modified/Normal Ratio
Protein N-terminal acetylation	42.0106	NSC34	Ala 2	Totally acetylated
		NSC34-SOD1WT		
		NSC34-SOD1G93A		
Oxidation	15.9949	NSC34	Met 155	5:1
		NSC34-SOD1WT		4:1
		NSC34-SOD1G93A		60:1
Dioxidation	31.9898	NSC34	Met 155	0.1:1
		NSC34-SOD1WT		0.1:1
		NSC34-SOD1G93A		5:1
Trioxidation	47.9847	NSC34	Cys 127	Totally trioxidized
		NSC34-SOD1WT	Cys 127	Totally trioxidized
		NSC34-SOD1G93A	Cys 127	30:1
Phosphorylation	79.9663	NSC34	Ser 104	0.01:1
		NSC34-SOD1WT		0.01:1
		NSC34-SOD1G93A		0.01:1
Deamidation	0.9840	NSC34	/	/
		NSC34-SOD1WT	/	/
		NSC34-SOD1G93A	Asn 37, 106, 207, 214, 239 Gln 166, 226	Asn = 0.01–0.6:1 Gln = deamidated in trace amount
